# Predicted Impacts of Booster, Immunity Decline, Vaccination Strategies, and Non-Pharmaceutical Interventions on COVID-19 Outcomes in France

**DOI:** 10.3390/vaccines10122033

**Published:** 2022-11-28

**Authors:** Simon Pageaud, Anne Eyraud-Loisel, Jean-Pierre Bertoglio, Alexis Bienvenüe, Nicolas Leboisne, Catherine Pothier, Christophe Rigotti, Nicolas Ponthus, Romain Gauchon, François Gueyffier, Philippe Vanhems, Jean Iwaz, Stéphane Loisel, Pascal Roy, on behalf of the CovDyn Group (Covid Dynamics)

**Affiliations:** 1 Université de Lyon, F-69000 Lyon, France; 2 Université Claude Bernard Lyon 1, F-69622 Lyon, France; 3 CNRS UMR 5558, Laboratoire de Biométrie et Biologie Évolutive, F-69100 Villeurbanne, France; 4 Service de Biostatistique-Bioinformatique, Pôle Santé Publique, Hospices Civils de Lyon, F-69003 Lyon, France; 5 Laboratoire de Sciences Actuarielle et Financière (LSAF), Institut de Science Financière et d’Assurances (ISFA), F-69007 Lyon, France; 6 Fondation du Risque, Groupe Louis Bachelier, F-75002 Paris, France; 7 CNRS UMR 5509, Laboratoire de Mécanique des Fluides et d’Acoustique (LMFA), F-69130 Écully, France; 8 École Centrale de Lyon, F-69130 Lyon, France; 9 Institut National des Sciences Appliquées de Lyon (INSA), F-69621 Villeurbanne, France; 10 CNRS UMR 5205, Laboratoire d’InfoRmatique en Image et Systèmes d’information (LIRIS), F-69100 Villeurbanne, France; 11 Centre INRIA de Lyon, F-69100 Villeurbanne, France; 12 CNRS UMR 5513, Laboratoire de Tribologie et Dynamique des Systèmes (LTDS), F-69130 Écully, France; 13 École Nationale des Travaux Publics de l’État (ENTPE), F-69120 Vaulx-en-Velin, France; 14 Service d’Hygiène, Épidémiologie, Infectiovigilance et Prévention, Hospices Civils de Lyon, F-69003 Lyon, France; 15 Centre International de Recherche en Infectiologie (CIRI), Inserm U1111, CNRS UMR 5308, École Nationale Supérieure de Lyon, F-69007 Lyon, France; 16 INSERM, F-CRIN, I-REIVAC (Réseau Innovative Clinical Research in Vaccinology), F-75004 Paris, France

**Keywords:** vaccination, COVID-19, agent-based model, decision support techniques, booster

## Abstract

The major economic and health consequences of COVID-19 called for various protective measures and mass vaccination campaigns. A previsional model was used to predict the future impacts of various measure combinations on COVID-19 mortality over a 400-day period in France. Calibrated on previous national hospitalization and mortality data, an agent-based epidemiological model was used to predict individual and combined effects of booster doses, vaccination of refractory adults, and vaccination of children, according to infection severity, immunity waning, and graded non-pharmaceutical interventions (NPIs). Assuming a 1.5 hospitalization hazard ratio and rapid immunity waning, booster doses would reduce COVID-19-related deaths by 50–70% with intensive NPIs and 93% with moderate NPIs. Vaccination of initially-refractory adults or children ≥5 years would half the number of deaths whatever the infection severity or degree of immunity waning. Assuming a 1.5 hospitalization hazard ratio, rapid immunity waning, moderate NPIs and booster doses, vaccinating children ≥12 years, ≥5 years, and ≥6 months would result in 6212, 3084, and 3018 deaths, respectively (vs. 87,552, 64,002, and 48,954 deaths without booster, respectively). In the same conditions, deaths would be 2696 if all adults and children ≥12 years were vaccinated and 2606 if all adults and children ≥6 months were vaccinated (vs. 11,404 and 3624 without booster, respectively). The model dealt successfully with single measures or complex combinations. It can help choosing them according to future epidemic features, vaccination extensions, and population immune status.

## 1. Background

The outbreak of SARS-CoV-2 virus is causing major national, European, and international crises with serious consequences in a large variety of domains. In the domain of health, the COVID-19 pandemic had deleterious consequences on public health (general morbidity and mortality) as on individual and social health. Several studies (recently cited or summarized by Barchielli et al. [1]) have been already dedicated to its impacts on mental health (e.g., cognitive decline), psychological well-being (stress, anxiety, depression, fears of contagion, death, possible vaccine consequences, etc.), social life (limited relationships, isolation), and behavior (changes in tobacco and alcohol consumption as in eating, sleeping, and exercise habits).

The COVID-19 pandemic started in China in late autumn 2019 and, on 31 December [2], the WHO China Office received reports on severe pneumonia cases in the city of Wuhan (Hubei Province). On 20 December 2021, around 280 million cases and 5.42 million deaths were recorded worldwide, of which 98 million cases and 1.67 million deaths in Europe and 8.8 million cases and 0.12 million deaths in France [3].

On December 14, 2020, the Alpha variant (lineage B.1.1.7) was identified in the United Kingdom [4]. In France, the first Alpha-related COVID-19 case was identified by the end of December 2020, and, later, on 8 June 2021, the variant was deemed responsible for 77% of diagnosed cases [5]. On October 2020, the Delta variant (lineage B.1.617.2) was identified in India with characteristics of higher infectiousness and increased severity (vs. Alpha) [6]. In France, the first Delta case was identified by the end of April 2021, and, later, on 22 June 2021, this variant was deemed responsible for 35.9% of diagnosed cases (and 99.6% of diagnosed cases on 21 September 2021). On November 2021, the Omicron variant (lineage B.1.1.529) was identified in Botswana. In Denmark, Ito et al [7] estimated the effective reproduction number of Omicron to be 3.19 times (95% CI: 2.82–3.61) greater than that of Delta. In France, the first Omicron-related COVID-19 case was identified in early December 2021, and, later, on 26 December 2021, the variant was deemed responsible for 44.7% of diagnosed cases (98.4% of cases on 21 January 2022). On 25 January, the peak of the 7-day moving average reached 366,179 contaminations [5]. The BA.2 sublineage became predominant on February 2022 [8] but was soon replaced by the BA.5 sublineage by the end of June 2022 [9].

In France, in 2020, two lockdown periods were decided to avoid saturation of intensive care unit (ICU) beds that would increase mortality. Precisely, the lockdown periods (17 March to 11 May and 30 October to 15 December 2020) aimed at limiting the magnitude of two successive epidemic waves.

In France, the dynamics of the epidemic is continuously assessed through changes in hospital admissions, ICU occupancy rate, and cumulative number of COVID-19-related deaths. Since the beginning of the pandemic in March 2020, protective measures have been constantly adapted to the dynamics of the epidemic. We have previously identified three levels of non-pharmaceutical interventions (NPIs) [10] and estimated their respective impacts on these dynamics. Moderate-NPIs (mask wearing and social distancing, but restaurants and leisure facilities still open) prevailed in summer and October 2020. More strict extended-NPIs (lockdown during weekends or full lockdown, as well as closure of schools, universities, and shopping facilities) prevailed in March-April 2020 and November 2020. Intensive NPIs (overnight curfew, social distancing, mask-wearing, increased distancing at work, closure of bars, restaurants and leisure facilities… but no strict lockdown) prevailed in December 2020–January 2021. Later, spring 2021 was a period of Extended NPIs, whereas summer 2021 was that of Moderate NPIs.

Anti-COVID vaccines have been rather rapidly developed. In 2020 and 2021, clinical trials reported that their efficacy in avoiding severe forms of the disease caused by the Alpha variant was 75% [11] and even 94% [12,13]. In France, a massive vaccination campaign (started on 27 December 2020), aimed to limit the occurrence of severe forms and increase herd immunity. It was first restricted to the most vulnerable people and health professionals. On June 2021, it became available to people aged ≥12 years and, on 22 December, to those aged ≥5 years. Vaccinating younger children is still not planned. On 1 September 2021, a booster dose (third injection) started to be recommended to the most vulnerable, then to all adults (≥18), then to all people (≥12) [14]. Now, the expected effects of this booster dose have to be determined in terms of ICU admission and COVID-19-related deaths.

One important public health issue is optimizing a vaccination schedule and adapting protective measures to the status of the epidemic and its potential changes. Obviously, the effectiveness of a vaccination campaign is highly dependent on the level of the epidemic, the prevalence of each variant, and the vaccination strategy; precisely, the booster dose administration, the vaccination of yet non-vaccinated adults, and the vaccination of children and young children. It has been recently observed that recontaminations associated with the waning of vaccine immunity are becoming increasingly frequent, despite complete vaccination schemes in a large majority of individuals [15].

The present simulation study aimed to predict COVID-19 mortality over a period of 400 days (23 November 2021 to 27 December 2022) on the basis of several scenarios that combined vaccination strategies, NPIs, and the waning of vaccine-conferred immunity over time. A previously-developed agent-based model [10] was extended to simulate, in addition to the effects of NPIs, the combined effects of a booster dose (third injection), complete vaccination of unvaccinated adults, vaccination of children aged 5 years and over, and vaccination of children aged 6 months and over.

## 2. Methods

### 2.1. Vaccination Scenarios Compared

Several scenarios have been developed and the results obtained with their various combinations displayed:

- *Immunity decrease*—Two scenarios were considered for two different speeds of immunity decrease over time. An ‘*optimistic* immunity decrease’ was supposed slower than a ‘*pessimistic* immunity decrease’.
- *Infection severity*—Two scenarios were considered: the same hospitalization ratio as with the Alpha variant (*hosp-same*) and a higher hospitalization ratio (*hosp-more*).
- *NPIs*—Three different NPI scenarios were considered: *moderate*, *intensive*, and *extended*.
- *Booster*—The model was developed with and without booster injection (*Booster vs. No Booster*).
- *New vacc/No new vacc*—Two scenarios were considered: previously non-vaccinated persons choose to receive a vaccine dose (*new-vacc*) or stay non-vaccinated (*no-new-vacc*).
- *Children vaccination*—Three scenarios were considered for children vaccination: that of children aged 12 years or more (≥12), that of children aged 5 years or more (≥5), and that of children aged 6 months or more (*All*).

### 2.2. The Agent-Based Model

The agent-based model (ABM) used in this work is an extension of the one previously designed to simulate the changes of the epidemic in France over 2021 and the first half of 2022 and compare the expected efficiencies of four theoretical vaccination campaigns combined with various NPIs [10].

**Figure 1:**
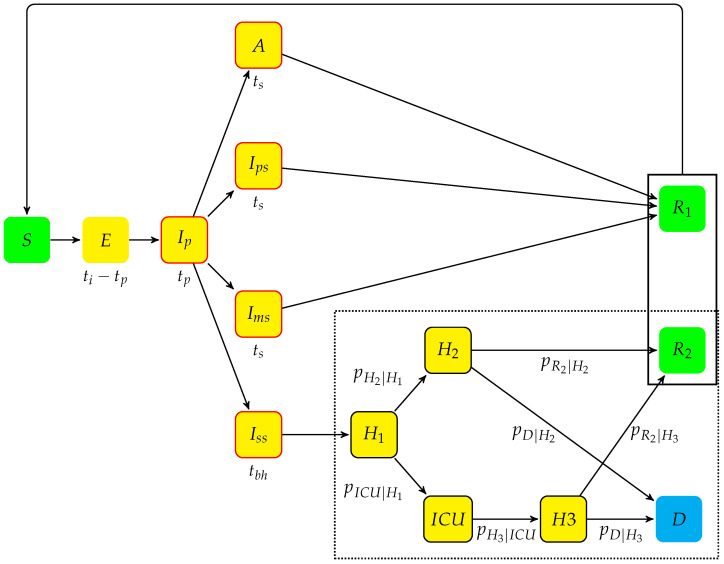
The states and their connections in the model. The left-hand part of the model represents the disease spreading with the average sojourn time beneath each compartment. The right-hand part of the model (bottom-right dotted frame) represents the hospitalized cases and their outcomes. It is composed of states $H_{1}$, $H_{2}$, $H_{3}$, ICU, $R_{2}$, and *D*. Each arrow is labeled with the individual daily transition probability between two states.

The previous ABM (Figure 1) extended the set of compartments initially proposed by Di Domenico et al. [16]; its left-hand side (that represents the ’disease spread’) followed the compartment model of Di Domenico et al., whereas its right-hand side (that represents the ’outcome of hospitalization’; dotted box in Figure 1) included specific states to account explicitly for hospitalization before and after periods of intensive care. Each state of the model considered nine age groups as in Gauchon et al. [17] and the transition probabilities—similar to those of a multi-state Markov model—were applied at individual level. The flexibility of the ABM allowed incorporating vaccination, variant prevalence, and NPIs, three aspects that were not handled by the model of Di Domenico et al. The parameters estimated or extracted from the literature [10] are displayed in Table 1 and Table 2. Here, the previous ABM has been extended to take into account the waning of the vaccine-conferred immunity or previous COVID-19 infection in susceptible or infected individuals. The additional arrows from residual states $R_{1}$ and $R_{2}$ to the susceptible state *S* characterize this extension. Based on this extended ABM, the simulations were carried out over a period of 400 days; precisely, from 23 November 2021 to 27 December 2022. Each simulation involved a sample of 645,000 agents (i.e., 1% of the French metropolitan population) with a distribution of age groups similar to that of the reference population. The simulation results were multiplied by 100 to obtain estimations for whole France. As these agent-based simulations are stochastic, the mean values and standard errors of the mean (SEM) across 50 runs were reported for each set of parameters.

#### 2.2.1. The ABM States

The model extended here is a stochastic ABM, with a discrete time step of one day.

- State *S* (susceptible) includes individuals who have never been infected with the virus or who became susceptible again after infection;
- The incubation period includes two states:
  − once infected, an individual moves from state *S* to state *E* (Exposed) that groups infected individuals who did not develop symptoms yet and are not contagious. The mean stay in *E* is $\left(t_{i}-t_{p}\right)$, $t_{i}$ being the incubation period and $t_{p}$ the duration of the prodromal state;
  − when an individual from *E* starts to be contagious, he or she is transferred to state $I_{p}$, a prodromal short phase that follows contamination, may not show symptoms but possible non-specific prodromes. After an average stay $t_{p}$, the individuals move to one of the four following states:
- *A* (Asymptomatic state, with probability $p_{a}$): individuals who completed the incubation period, became infectious, but do not show disease symptoms. The mean stay in *A* is $t_{s}$;
- The symptomatic infectious period includes three states for individuals who develop symptoms (with probability $1-p_{a}$):
  − $I_{ps}$ (Paucisymptomatic disease): individuals with weak disease symptoms;
  − $I_{ms}$ (Medium symptoms): individuals with disease symptoms (e.g., fever or cough) who do not require hospitalization. The average stays in states $I_{ps}$ and $I_{ms}$ are the same as in state *A*;
  − $I_{ss}$ (Severe symptoms): severely infected individuals that require hospitalization. These stay in $I_{ss}$ before hospitalization. The mean stay in $I_{ss}$ is $t_{bh}$.
  Once symptoms are present, the probabilities of being in states $I_{ps}$, $I_{ms}$, and $I_{ss}$ are respectively $p_{I_{ps}}$, $p_{I_{ms}}$, and $p_{ss}$ (probabilities summing to one).
  An individual who leaves state *A*, $I_{ps}$, or $I_{ms}$ ends in state $R_{1}$ (Individuals removed, with possibility of re-infection).
- After leaving $I_{ss}$, the individuals enter a hospitalization period, which corresponds to one of the four following states:
  − $H_{1}$: individuals hospitalized before stating whether they need intensive care or not;
  − $H_{2}$: individuals hospitalized without need for intensive care;
  − ICU: individuals hospitalized in an intensive care unit;
  − $H_{3}$: individuals hospitalized after leaving an ICU.
  After hospitalization, individuals go to either one of two states:
  − *D*: individuals deceased at hospital, an `absorbing’ state;
  − $R_{2}$: individuals removed, with the possibility of being re-infected.
  During hospitalization, each individual follows a Markov chain dynamics, with daily transition probabilities noted $p_{H_{2}|H_{1}}$, $p_{ICU|H_{1}}$, $p_{H_{3}|ICU}$, $p_{D|H_{2}}$, $p_{R_{2}|H_{2}}$, $p_{D|H_{3}}$, and $p_{R_{2}|H_{3}}$.

The age distribution of the population was obtained from the Institut National de la Statistique et des Études Économiques. Nine age groups were considered: eight ten-year age groups from 0 to 79 years plus an extra age-group with individuals aged 80 or older. The contact matrix was available for France on the basis of these age ranges [20]. The number of daily contacts per individual was set at the beginning of the simulation and drawn from a Poisson distribution whose parameter was provided by the contact matrix. At each time step, the daily contacts between infectious and susceptible individuals were random.

#### 2.2.2. The Transition Probabilities

The disease spreads by infectious contacts that move individuals from state S to state E. Daily contacts between individuals were based on a contact matrix *C*, $C_{i,j}$ being the daily average number of individuals of age group *j* encountered by individuals of age group *i*. $C_{i.}=\sum_{j}C_{i,j}$ was the daily average number of individuals encountered by individuals of age group *i*. The daily number of individuals encountered by a new individual was obtained from a Poisson distribution with parameter $C_{i.}$, the expected proportions of contacts aged *j* being $C_{i,j}$/$C_{i.}$.

The daily probability $P_{\mathrm{infect}}$ (that of a susceptible individual to be infected by contact with an infectious individual) was decomposed into a product of six terms (multiplicative model):

(1) $$\begin{array}{c} P_{\mathrm{infect}}\left(\mathrm{age},\mathrm{NPI},\mathrm{strain},Z,\delta_{I},\delta_{S},t_{I}\right)=\;\\ \;\beta_{1,\mathrm{age}}\;\beta_{2,\mathrm{NPI}}\;\beta_{3,\mathrm{strain}}i_{Z}{\left(1-\beta_{4_{1}}\right)}^{\delta_{I}}{\left(1-\beta_{5_{1}}\beta_{5_{2}}\left(t_{I}\right)\right)}^{\delta_{S}} \end{array}$$

- $\beta_{1,\mathrm{age}}$ is the estimated baseline daily probability of infection as a function of the age of the susceptible individual (after a contact with an infectious individual).
- $\beta_{2,\mathrm{NPI}}$, NPI∈{Moderate,Intensive,Extended}, estimated the effects of three NPI levels: moderate, intensive and extended. The underlying hypotheses were that the NPI effects are not dependent on the state of the infectious individual, the age group, or the virus strain. Parameter $\beta_{2,\mathrm{NPI}}$, was interpreted as a coefficient that reduces the mean number of daily contacts, and/or the mean duration of contact, and/or contact infectiousness ($0\leq \beta_{2,\mathrm{NPI}}\leq 1$). The three NPI levels were fixed at values estimated in a previous study: $\beta_{2,\mathrm{Moderate}}=1$, $\beta_{2,\mathrm{Intensive}}=0.783$, and $\beta_{2,\mathrm{Extended}}=0.534$ [10].
- $\beta_{3,\mathrm{strain}}$, strain∈{orginal,Alpha,Delta}, estimated the relative contagiousness of each virus strains vs. the original strain ($\beta_{3,\mathrm{original}}=1$). The relative contagiousness of the Alpha variant was previously estimated at $\beta_{3,\mathrm{Alpha}}=1.572$ [10]. Assuming the Delta variant to be 60% more contagious than Alpha [6,21], $\beta_{3,\mathrm{Delta}}\approx 1.6\times \beta_{3,\mathrm{Alpha}}=1.6\times 1.572\approx 2.515$.
- $Z\in \{E,I_{p},A,I_{ps},I_{ms},I_{ss}\}$ is the state of an infectious individual; $i_{Z}$ being the relative infectiousness of individuals in state *Z*. The proportion of asymptomatic forms was set to 20% (Table 1). The relative infectiousness of the prodromal, asymptomatic, and paucisymptomatic forms were set to $i_{I_{p}}=i_{A}=i_{I_{ps}}=0.55$, the medium and severe symptom forms being taken as references with $i_{I_{ms}}=i_{I_{ss}}=1$ according to the literature (Table 1). The stays in states *E*, $I_{p}$, *A*, $I_{ps}$, $I_{ms}$, and $I_{ss}$ were generated from Weibull distributions. When more than one destination state was possible, the transition was selected at random using the corresponding probability (i.e., $p_{A}$, $p_{I_{ps}}$, $p_{I_{ms}}$ or $p_{I_{ss}}$). The proportions of the various symptomatic forms were considered dependent on age (Table 2).
- The reduction in virus transmission due to the vaccinated status (or previous COVID-19) of infectious individuals was modelled as follows. Indicator $\delta_{I}$ characterized the immune status of the infecting individual who had received a vaccine or had COVID-19 at least 7 days before ($\delta_{I}=1$; otherwise $\delta_{I}=0$). Parameter $\beta_{4_{1}}$ was the reduction in virus transmission.
- The reduction in virus transmission associated with a vaccinated status (or previous COVID-19) of susceptible individuals was modelled as follows. Indicator $\delta_{S}$ characterized the immune status of susceptible individual who received a vaccine injection or had COVID-19 at least 7 days before ($\delta_{S}=1$; otherwise $\delta_{S}=0$). Parameter $\beta_{5_{1}}$ was the initial vaccine-related reduction in virus transmission. Parameter $\beta_{5_{2}}\left(t_{I}\right)$, whose value decreased as a function of $t_{I}$, the time elapsed after the last injection, characterized the progressive decrease of this reduction over time.

In the right-hand part of the model (dotted frame, Figure 1), on each day spent by an individual in state $H_{1}$, $H_{2}$, $H_{3}$, or ICU, a state transition was drawn with probabilities $p_{H_{2}|H_{1}}$, $p_{ICU|H_{1}}$, $p_{H_{3}|ICU}$, $p_{D|H_{2}}$, $p_{D|H_{3}}$, $p_{R_{2}|H_{2}}$, or $p_{R_{2}|H_{3}}$. Staying in the same state (i.e., a self-loop) was considered a complementary event.

#### 2.2.3. The Left-Hand Side of the Model (Figure 1)

- All new injections were considered given with a mRNA vaccine.
- Simulations were performed with each of the three NPI levels whose parameters were previously estimated at $\beta_{2,\mathrm{Moderate}}=1$, $\beta_{2,\mathrm{Intensive}}=0.783$, and $\beta_{2,\mathrm{Extended}}=0.534$.
- The effect of an additional booster dose of vaccine in not-previously infected individuals (third injection) or in previously infected individuals (second injection) was estimated by comparing simulation results obtained with and without this booster dose. This injection was considered to be given five months after the previous one (or after infection). For convenience, all previously vaccinated individual were supposed given this booster dose.
- The effect of complete vaccination of refractory individuals was estimated by comparing simulation results obtained with and without complete vaccination of these individuals. For convenience, complete vaccination was supposed to be achieved within one month.
- The expected effect of children vaccination was estimated by comparing the results obtained with vaccination of only individuals ≥12 years (i.e., 12 to 18), all children ≥5 years (i.e., 5 to 18), and all children ≥6 months (i.e., 6 months to 18 years). For convenience, the vaccination of children was supposed to be achieved within six months.
- The reduction in virus transmission due to the vaccinated status (or to a history of COVID-19) in the infectious individuals was fixed at $\beta_{4_{1}}=0.50$.
- The reduction in virus transmission in the susceptible individuals after the first injection (or first infection) was fixed at $\beta_{5_{1}}=0.80$ without linear decrease before the second injection (3 weeks later) or the occurrence of COVID-19. The reduction in susceptible individuals after the second injection (or infection following the first dose of vaccine) was fixed at $\beta_{5_{1}}=0.80$, and its linear decrease six months later ($\beta_{5_{2}}\left(t_{I}\right)$) set so as to obtain a value of 0.58 (optimistic immunity decline) or 0.44 (pessimistic immunity decline). A similar approach was proposed by Bosetti et al. [22]. The reduction in virus transmission due to the booster dose of vaccine in never-infected individuals (third injection) or in previously infected then susceptible again individuals (second injection) was fixed at $\beta_{5_{1}}=0.95$ without decrease.

#### 2.2.4. The Right-Hand Side of the Model (Figure 1)

- Two levels of infection severity were considered for the Delta variant: either similar to the severity of the Alpha variant (hosp-same) $p_{I_{ss},\delta }=p_{I_{ss},\alpha }$, or higher with a hazard ratio of hospitalization of 1.5 (hosp-more) $p_{I_{ss},\delta }=1-{\left(1-p_{I_{ss},\alpha }\right)}^{1.5}$.
- The reduction in the severity of infection after vaccination was specified as follows. It combined, after a second injection of vaccine in previously uninfected individuals (or after a single injection in previously infected individuals), an initial 95% reduction in the risk of hospitalization with a linear decrease of this reduction so as to obtain a value of 0.88 or 0.78 six months later with optimistic and pessimistic immunity decrease, respectively. After a booster dose of vaccine in previously uninfected individuals (third injection) or in previously infected individuals (second injection), a 95% reduction in the risk of hospitalization was set without change over time.

## 3. Results

### 3.1. Immunity Decrease, Infection Severity, and NPIs

Table 3 shows the results of simulations in case all previously vaccinated persons receive a booster dose, all initially vaccine-refractory persons still refuse the vaccine, and children under 12 years of age are not vaccinated. With intensive vs. moderate NPIs, the number of deaths would be at least halved, but, with extended vs. intensive NPIs, the additional decrease would be 11 to 20%. With moderate NPIs, more deaths would occur in case of higher infection severity. The effect of infection severity would be attenuated if intensive or extended NPIs were applied. Similar results would be seen whatever the degree of immunity decrease.

### 3.2. The Booster Effect

The impact of a booster dose if initially vaccine-refractory persons still refuse the vaccine and children under 12 are not vaccinated is shown in Table 4. With moderate NPIs, the booster would divide the number of deaths by 6.6 (38,146 down to 5760) in the most favorable scenario (hospitalization hazard ratio of 1 and “optimistic immunity decrease”) but by 14 (87,552 down to 6212) in the worst scenario (hospitalization hazard ratio of 1.5 and “pessimistic immunity decrease”). A shift from an optimistic to a pessimistic immunity decrease would lead to a rather similar number of deaths with the booster but to twice that number without the booster. Without booster, an increased disease severity would be associated with a 12% increase in mortality (from 38,146 to 42,850 deaths) in an optimistic immunity decrease condition but a 16% increase in mortality (from 75,766 to 87,552 deaths) in a pessimistic immunity decrease condition.

With intensive vs. moderate NPIs and booster administration, the number of deaths would be divided by slightly more than two, whereas without a booster, it would be divided by nearly 10. With extended vs. intensive NPIs, an additional decrease of 11 to 20% would be seen with a booster but 45 to 72% decrease without a booster.

### 3.3. Complete Vaccination of All Adult Population

Without booster, the effect of vaccinating all adults would be impressive: the number of deaths would be divided by 8 (87,552 down to 11,404) in the worst scenario (no booster, pessimistic immunity decrease, and increased disease severity).

Other simulated scenarios with and without vaccination of initially vaccine-refractory adults gave the results displayed in Table 5. With booster and moderate NPIs, acceptance of vaccination by these adults would half the number of deaths whatever the decrease in immunity or infection severity. A much smaller reduction, by less than 10%, would be seen with intensive or extended NPIs.

### 3.4. Vaccination of Young and Very Young Children

The study sought for the results of simulated scenarios in which vaccinated individuals receive a booster and children are vaccinated starting from different minimum ages (Table 6). With moderate NPIs, vaccinating children aged 5 and over would divide the number of deaths by almost 2 whatever the immunity decrease or the infection severity, whereas vaccinating children ≥6 months would result in a +5% reduction in the number of deaths. With intensive or extended NPIs, the effect of vaccinating children under 12 would be much smaller or stay within the limits of result fluctuations.

### 3.5. Comparing Separate Effects

Without a booster and with a pessimistic immunity decrease, an increased infection severity, and moderate NPIs, the number of deaths would be 87,552 if children ≥12 years were vaccinated (Table 4), 64,002 if children ≥5 years were vaccinated, and 48,954 if children ≥6 months were vaccinated. In the same conditions, the number of deaths would be 11,404 if all adults and children ≥12 years were vaccinated, and 3624 if all adults and children ≥6 months were vaccinated. Vaccinating all adults would result in much less deaths than vaccinating children <12 years.

With a booster and with a pessimistic immunity decrease, an increased infection severity, and moderate NPIs, the number of deaths would be 6212 if children ≥12 years were vaccinated (Table 4), 3084 if children ≥5 years were vaccinated, and 3018 if children ≥6 months were vaccinated (Table 6). In the same conditions, the number of deaths would be 2696 if all adults and children ≥12 years were vaccinated (Table 5) and 2606 if all adults and children ≥6 months were vaccinated.

The booster would then often lead to an impressive decrease in the number of deaths. The additional effect of vaccinating all adults would be slightly higher than that of vaccinating all children.

## 4. Discussion

Controlling the spread of COVID-19 included first personal and group non-pharmacological prevention measures (mask wearing, hands washing, social distancing, home isolation, curfews, lockdowns, travel and gathering restrictions, etc.) [23]. When anti-COVID-19 vaccines became available, vaccination campaigns were organized for specific age groups then extended to others–but not yet all. Meanwhile, hardly acceptable intensive or extended NPIs had to be eased to resume social and economic activities. Vaccination refusals, the emergence of fast-spreading variants, the waning of vaccine-conferred immunity over a few months and the need for booster injections further complicated the epidemiological situation. Previsional models became essential to estimate the impacts of single, but more importantly, combined measures either on the disease spread or on its outcomes (morbidity and mortality) and help choosing the most efficient strategy given local epidemiological, demographic, and virus-related data.

A previously published stochastic agent-based model [10] has estimated that “with the sole emergence of Alpha variant of the COVID-19 virus, it is more than 600,000 deaths in France that would have been observed without vaccination, even under strict barrier measures”. The present simulation model extends the previous model and uses previously estimated NPI effects to predict the combined effects of a booster dose of vaccine (3rd injection), vaccination of all unvaccinated adults, vaccination of children aged ≥5 years, and vaccination of children aged ≥6 months.

The left-hand side of the model (Section 2.2, Figure 1) included the determinants of virus transmission (age, NPIs, virus strain, immune status of infected and susceptible individuals) and its right-hand side the determinants of the probability to develop a severe or fatal form of the disease. The proportion of the adult population that complied with the proposed vaccination program was also taken into account as a key determinant of controlling the epidemic and its associated mortality.

The retained relative contagiousness of the Delta variant was 1.6 times that of the Alpha variant; thus, 2.5 times that of the original strain [6,21]. Without booster and with a decrease in vaccine immunity over time, the resumption of the epidemic could be due to a higher contagiousness of the Delta variant within a context of moderate NPIs; whereas a better control of the epidemic would be obtained within a context of intensive NPIs ($\beta_{2,\mathrm{Intensive}}=0.783$) or extended NPIs ($\beta_{2,\mathrm{Extended}}=0.534$). Given the multiplicative combined effects of the determinants of viral transmission, the protection provided by intensive NPIs counterbalanced only partially the higher contagiousness of the Delta variant vs. the Alpha variant.

The results of the immunity coverage provided by the booster dose was considerable. The assumptions of vaccine-conferred immunity waning after the initial vaccination and maintenance of a protective immunity after the booster may have favored these results. The assumptions are consistent with the current knowledge about the need for a booster to achieve prolonged vaccine-conferred immunity. If poeple ≥12 years are vaccinated, without vaccination of initially vaccine-refractory adults, a booster dose would divide the number of deaths by 6.6 to 14 according to disease severity and degree of immunity waning. The herein simulation results were fully consistent with those of other epidemiological studies or clinical trials. In a case-control study involving a pharmacy screening programme in 49 US states, the receipt of three doses of mRNA vaccine conferred a higher protection against symptomatic SARS-CoV-2 infection by the Delta variant than the receipt of two doses (adjusted OR = 0.16, 95% CI: 0.14–0.17) [24]. In a multicentre randomized placebo-controlled clinical trial (July 1 to August 10, 2021) that involved 10,136 individuals previously-vaccinated with two doses of BNT162b2, the vaccine booster prevented 95.3% (95% CI: 89.5–98.3%) of cases two months after this third injection [25].

The effect of vaccinating all adults would be impressive: without booster and moderate NPIs, the number of deaths would be divided by 8 in the worst scenario (pessimistic immunity decrease and increased disease severity). This number would be halved with booster and moderate NPIs whatever the decrease in immunity or infection severity. In the same context, vaccinating children ≥5 years would also half the number of deaths. Thus, it would be more advantageous if initially vaccine-refractory adults could accept vaccination (to reduce their own risk of developing a severe disease) versus vaccinating young children (to protect unvaccinated adults via limiting virus circulation). Nevertheless, this should not hinder children vaccination (to develop herd immunity).

One interest of the proposed modelling is the possibility of identifying parameters that can be adjusted to limit the spread of the epidemic and related mortality. Many disease-fighting scenarios may be developed by anticipating possible changes in variant contagiousness, infection severity, and vaccine-conferred immunity. The scenarios compared above were based on previous calibration of some parameters relative to the original virus and its Alpha variant but took into account the higher contagiousness of the Delta variant. The proposed model assumes multiplicative effects of the age-specific baseline daily probability of infection, the prevention measures (NPIs), the virus strain, and the decline in immunity over time on the infection daily probability of a susceptible person after contact with an infectious one $P_{\mathrm{infect}}$. More complex models may be developed through introducing interaction terms between the model parameters. A strictly multiplicative model was retained at this stage of the simulations, given available estimated parameters.

The proposed model was developed before the emergence of the Omicron variant; however, it can be easily adapted to various degrees of variant contagiousness and disease aggressiveness. The parameters related to vaccine efficacy and conferred immunity may be also adapted. A case-control study that evaluated the efficacy of a BNT162b2 vaccine (two doses) against the Omicron variant has found a limited protection against a symptomatic disease 20 weeks after the second injection, whereas a booster dose was found associated with a significantly higher protection despite immunity waning over time [26].

The proposed model may also help studying the long-term impacts of booster injection frequencies, according to the characteristics of a given population. Using the Israeli Ministry of Health database, a study conducted between 10 January and 2 March 2022 (when Omicron was most prevalent) has confirmed that both the incidence and the severity of SARS-Cov-2 infection were lower after a fourth dose of BNT162b2 vaccine than after only three doses [27].

In the simulations carried out here, strategies were compared without constraint relative to the access to the vaccine. Thus, vaccinating either all still-refractory adults or children <12 years was not supposed to slow down the administration of booster shots. This choice is justified, at least in France, because of the currently high vaccination coverage (53.7 million French people as of 7 September 2022, of which 40.6 millions have received a booster dose: i.e., respectively, 79.2 and 59.9% of the whole French population). With a variant as contagious as Omicron, other results would be obtained in case of limited access to vaccination; e.g., prioritising one immunisation strategy would penalise another by slowing down the delivery of vaccines to the latter.

One of the main difficulties in studying the epidemiology of COVID-19 is the lack of descriptive data needed to estimate the extent of the virus spread at a national level. Indeed, data stemming from analyses of severe or fatal forms of the disease provided only a proxy of the overall spread of the epidemic. The data on positive RT-PCR or antigenic tests provide only the minimum value of the prevalence of the infection at a given time and the changes in test positivity over time lead to biased estimates of the prevalence. In France, more than 270 million RT-PCR and antigenic tests were performed over nearly two years (mid-March 2020 to end of February 2022), a peak of 2.2 million tests being reached on 10 January 2022 [28]. Given the number of tests performed, it is regrettable that no representative weekly random-sample epidemiological follow-up study has been conducted. The paucity of available descriptive epidemiological data imposed the development of complex models, without providing data necessary for their optimization. It is therefore essential to anticipate the implementation of high-quality descriptive epidemiological studies to allow monitoring future epidemic waves and assessing the impact of any control measures.

## 5. Conclusions

In the absence of other preventive solutions, lockdowns (extended NPIs) were necessary to stop the first epidemic waves. Lockdowns seem now quite obsolete given the availability of vaccines that represent the most effective control measure for epidemics spreads and their related complications. More than two years after the start of the epidemic, only moderate NPIs seemed acceptable to help an effective restart of economic and social activities. It seems therefore appropriate to optimize the vaccination strategies to avoid the need for intensive or extended NPIs.

Given the parameters kept for the simulations, the booster dose appeared to be highly effective against the Delta variant and the proposed simulation approach allowed a better understanding of the combined effects of various determinants of the disease. As the COVID-19 epidemic is not yet under full control, the proposed approach could be useful for planning future vaccination campaigns. Representative data on the prevalence of infection are needed to anticipate rapid changes in disease patterns, so that the results of analyses of these data can be combined with the available results of other analytical studies and clinical trials.

There is no doubt that by refining and, above all, simplifying the use of such a model (despite its inherent complexity), health authorities will have a tool capable of analysing local or national situations and advising on the most effective decisions for epidemic control.

## Figures and Tables

**Table 1:** The model parameters as extracted from the literature (extended from [10]).

Parameter	Value	Reference
*Sojourn time*		
$t_{i}$	5.1	see [17]
$t_{p}$	1.5	see [17]
$t_{s}$	7	see [17]
$t_{bh}$	3	see [17]
$t_{R_{1}}$	60	
$t_{R_{2}}$	60	
*Relative infectiousness without vaccine*		
$i_{Ims}$	1	[18]
$i_{Iss}$	1	[18]
$i_{A}$	0.55	[18]
$i_{Ip}$	0.55	[18]
$i_{Ips}$	0.55	[18]
Rate of asymptomatic subjects $p_{A}$	0.20	[16,19]
Proportion of Delta variant (21 September 2021)	99.6%	Santé Publique France

**Table 2:** Estimated parameters of the left- and right-hand parts of the model (extracted and modified from [10]).

	All Ages	0–9	10–19	20–29	30–39	40–49	50–59	60–69	70–79	≥80
Left-hand side										
$\beta_{1,\mathrm{age}}$		0.01	0.01	0.017	0.008	0.007	0.012	0.016	0.16	0.164
$p_{I_{ps}}$		0.249	0.249	0.247	0.243	0.242	0.225	0.209	0.189	0.031
$p_{I_{ms}}$		0.746	0.748	0.741	0.730	0.727	*0.674*	0.626	0.568	0.092
$p_{I_{ss}}$		0.006	0.003	0.012	0.026	0.031	0.102	0.166	0.243	0.877
$\beta_{2,\mathrm{Intensive}}$	0.783									
$\beta_{2,\mathrm{Extended}}$	0.534									
$\beta_{3,\mathrm{Alpha}}$	1.572									
$\beta_{3,\mathrm{Delta}}$	2.500									
Right-hand side										
$p_{ICU|H_{1}}$		0.052	0.031	0.077	0.071	0.116	0.157	0.280	0.268	0.098
$p_{H_{2}|H_{1}}$		0.948	0.967	0.902	0.918	0.873	0.841	0.396	0.732	0.895
$p_{H_{3}|ICU}$		0.079	0.033	0.070	0.033	0.033	0.033	0.075	0.057	0.139
$p_{D|H_{2}}$		0	0	0.001	0.001	0.002	0.003	0.009	0.012	0.016
$p_{R_{2}|H_{2}}$		0.288	0.193	0.165	0.151	0.115	0.082	0.040	0.035	0.030
$p_{D|H_{3}}$		0	0	0.001	0.005	0.004	0.007	0.001	0.004	0.014
$p_{R_{2}|H_{3}}$		0.084	0.052	0.211	0.057	0.055	0.053	0.102	0.059	0.073

**Table 3:** Number of deaths ±SEM over 400 days in hypothetical combinations of non-pharmaceutical intervention levels, immunity decrease, and infection severity.

Immunity and Infection Severity	Non-Pharmaceutical Intervention		
	Moderate	Intensive	Extended
Optimistic immunity decrease			
$PI_{ss},\delta =PI_{ss},\alpha {\;}^{*}$	5760±89	2502±65	2004±52
$PI_{ss},\delta >PI_{ss},\alpha {\;}^{†}$	6350±106	2444±47	2164±59
Pessimistic immunity decrease			
$PI_{ss},\delta =PI_{ss},\alpha {\;}^{*}$	5900±101	2540±66	2040±48
$PI_{ss},\delta >PI_{ss},\alpha {\;}^{†}$	6212±114	2534±57	2166±58

Children ≥ 12 are vaccinated. All vaccinated individuals receive a booster dose of vaccine. Vaccine-refractory adults are still not vaccinated. * Equal probabilities of being hospitalized with Alpha than with Delta variant. ^†^ Higher probability of being hospitalized with Delta than with Alpha variant.

**Table 4:** Number of deaths ±SEM over 400 days in hypothetical combinations of non-pharmaceutical intervention levels, immunity decrease, and infection severity, with and without a booster dose of vaccine given to all previously vaccinated individuals.

	Non-Pharmaceutical Intervention					
Immunity and Infection Severity	Moderate		Intensive		Extended	
	With Booster	No Booster	With Booster	No Booster	With Booster	No Booster
Optimistic immunity decrease						
$PI_{ss},\delta =PI_{ss},\alpha {\;}^{*}$	5760±89	38,146±361	2502±65	3908±90	2004±52	2162±54
$PI_{ss},\delta >PI_{ss},\alpha {\;}^{†}$	6350±106	42,850±371	2444±47	3966±97	2164±59	2162±54
Pessimistic immunity decrease						
$PI_{ss},\delta =PI_{ss},\alpha {\;}^{*}$	5900±101	75,766±385	2540±66	7532±190	2040±48	2142±53
$PI_{ss},\delta >PI_{ss},\alpha {\;}^{†}$	6212±114	87,552±524	2534±57	7712±244	2166±58	2338±67

Children ≥ 12 are vaccinated. Vaccine-refractory adults are still not vaccinated. * Equal probabilities of being hospitalized with Alpha than with Delta variant. ^†^ Higher probability of being hospitalized with Delta than with Alpha variant.

**Table 5:** Number of deaths ±SEM over 400 days in hypothetical combinations of non-pharmaceutical intervention levels, immunity decrease, and infection severity, with and without new vaccinations (that of refractory adults).

	Non-Pharmaceutical Intervention					
Immunity and Infection Severity	Moderate		Intensive		Extended	
	New Vaccinations	No New Vaccinations	New Vaccinations	No New Vaccinations	New Vaccinations	No New Vaccinations
Optimistic immunity decrease						
$PI_{ss},\delta =PI_{ss},\alpha {\;}^{*}$	2612±68	5760±89	2302±55	2502±65	2032±55	2004±52
$PI_{ss},\delta >PI_{ss},\alpha {\;}^{†}$	2686±73	6350±106	2368±55	2444±47	2016±60	2164±59
Pessimistic immunity decrease						
$PI_{ss},\delta =PI_{ss},\alpha {\;}^{*}$	2624±51	5900±101	2346±70	2540±66	1994±58	2040±48
$PI_{ss},\delta >PI_{ss},\alpha {\;}^{†}$	2696±60	6212±114	2332±59	2534±57	2082±50	2166±58

Children ≥ 12 years are vaccinated. All vaccinated individuals receive a booster dose of vaccine. * Equal probabilities of being hospitalized with Alpha than with Delta variant. ^†^ Higher probability of being hospitalized with Delta than with Alpha variant.

**Table 6:** Number of deaths ±SEM over 400 days in hypothetical combinations of non-pharmaceutical intervention levels, immunity decrease, and infection severity, with vaccinating children ≥12 years, ≥5 years, or all children ≥6 months (All).

Immunity and Infection Severity	Non-Pharmaceutical Intervention								
	Moderate			Intensive			Extended		
	≥12 Years	≥5 Years	All	≥12 Years	≥5 Years	All	≥12 Years	≥5 Years	All
Optimistic immunity decrease									
$PI_{ss},\delta =PI_{ss},\alpha {\;}^{*}$	5760±89	3084±68	2890±64	2502±65	2454±56	2418±55	2004±52	2102±49	2178±65
$PI_{ss},\delta >PI_{ss},\alpha {\;}^{†}$	6350±106	3222±82	3006±79	2444±47	2526±61	2464±58	2164±59	2058±60	2156±53
Pessimistic immunity decrease									
$PI_{ss},\delta =PI_{ss},\alpha {\;}^{*}$	5900±101	3090±73	2998±85	2540±66	2444±63	2398±69	2040±48	2076±64	1974±56
$PI_{ss},\delta >PI_{ss},\alpha {\;}^{†}$	6212±114	3084±73	3018±74	2534±57	2482±64	2528±63	2166±58	2094±56	2112±47

Vaccinated individuals receive a booster dose of vaccin. Vaccine-refractory adults are still not vaccinated. * Equal probabilities of being hospitalized with Alpha than with Delta variant. ^†^ Higher probability of being hospitalized with Delta than with Alpha variant.

## Data Availability

All data generated during this study are available from the corresponding author on reasonable and motivated request. The prevalence values in France are publicly available from Santé Publique France https://www.santepubliquefrance.fr/ (accessed on 3 October 2022) for Alpha, Delta, and Omicron variants. The data used for estimation are also publicly available from Santé Publique France https://www.data.gouv.fr/fr/datasets/donnees-hospitalieres-relatives-a-lepidemie-de-covid-19/ (accessed on 3 October 2022). The prevalence of Removed individuals in France on 1 January 2021, are publicly available from Institut Pasteur https://modelisation-covid19.pasteur.fr/realtime-analysis/infected-population/ (accessed on 3 October 2022).

## Appendix A. Members of the CovDyn (Covid Dynamics) Group, by Main Affiliation

LBBE—HCL

Mathieu FAUVERNIER, François GUEYFFIER, Jean IWAZ, Delphine MAUCORT-BOULCH, Simon PAGEAUD, Muriel RABILLOUD, Pascal ROY.

LSAF

Alexis BIENVENÜE, Anne EYRAUD-LOISEL, Romain GAUCHON, Pierre-Olivier GOFFARD, Nicolas LEBOISNE, Stéphane LOISEL, Xavier MILHAUD, Denys POMMERET, Yahia SALHI.

LAMA

Pierre VANDEKERKHOVE.

CIRI- HCL

Philippe VANHEMS.

LMFA

Jean-Pierre BERTOGLIO.

LTDS

Nicolas PONTHUS.

INL

Christelle YEROMONAHOS.

LIRIS

Stéphane DERRODE, Véronique DESLANDRES, Mohand-Saïd HACID, Salima HASSAS, Catherine POTHIER, Christophe RIGOTTI.

ICJ

Thibault ESPINASSE, Samuel BERNARD, Philippe MICHEL, Vitaly VOLPERT.

INRIA

Mostafa ADIMY.

CHU De Rouen Unité INSERM 1018, CESP

Jacques BENICHOU.

CHU De Rouen LIMICS INSERM U1142, Université de Rouen/ Sorbonne Université

Stéfan DARMONI.

CHU de NICE, Université de Nice

Pascal STACCINI.
